# Building and sustaining public and political commitment to the value of vaccination: Recommendations for the Immunization Agenda 2030 (Strategic Priority Area 2)^☆^

**DOI:** 10.1016/j.vaccine.2022.11.038

**Published:** 2024-04-08

**Authors:** Folake Olayinka, Molly Sauer, Lisa Menning, Diane Summers, Chizoba Wonodi, Susan Mackay, Noni E. MacDonald, J. Peter Figueroa, Benjamin Andriamitantsoa, George Bonsu, Pradeep Haldar, Ann Lindstrand, Lora Shimp, for the IA2030 Strategic Priority 2 Commitment, Demand Working Group

**Affiliations:** a JSI Research & Training Institute, Inc, Arlington, USA; b Department of International Health, Johns Hopkins Bloomberg School of Public Health, Baltimore, USA; c International Vaccine Access Center, Johns Hopkins Bloomberg School of Public Health, Baltimore, USA; d Department of Immunization, Vaccines and Biologicals, World Health Organization, Geneva, Switzerland; e UNICEF, NY, USA; f Gavi, the Vaccine Alliance, Geneva, Switzerland; g Dalhousie University, Halifax, Canada; h University of the West Indies, Kingston, Jamaica; i Freelance Consultant; Parliament of Madagascar, Antananarivo, Madagascar; j Expanded Program on Immunization, Government of Ghana, Accra, Ghana; k Ministry of Health and Family Welfare, Government of India, New Delhi, India

**Keywords:** Vaccination, Demand, Commitment, Acceptance

## Abstract

Vaccines have contributed to substantial improvements in health and social development outcomes for millions in recent decades. However, equitable access to immunization remains a critical challenge that has stalled progress toward improving several health indicators around the world. The COVID-19 pandemic has also negatively impacted routine immunization services around the world further threatening universal access to the benefits of lifesaving vaccines. To overcome these challenges, the Immunization Agenda 2030 (IA2030) focuses on increasing both commitment and demand for vaccines. There are three broad barriers that will need to be addressed in order to achieve national and subnational immunization targets: (1) shifting leadership priorities and resource constraints, (2) visibility of disease burden, and (3) social and behavioral drivers. IA2030 proposes a set of interventions to address these barriers. First, efforts to ensure government engagement on immunization financing, regulatory, and legislative frameworks. Next, those in subnational leadership positions and local community members need to be further engaged to ensure local commitment and demand. Governance structures and health agencies must accept responsibility and be held accountable for delivering inclusive, quality, and accessible services and for achieving national targets. Further, the availability of quality immunization services and commitment to adequate financing and resourcing must go hand-in-hand with public health programs to increase access to and demand for vaccination. Last, strengthening trust in immunization systems and improving individual and program resilience can help mitigate the risk of vaccine confidence crises. These interventions together can help ensure a world where everyone, everywhere has access to and uses vaccines for lifesaving vaccination.

## 1 Introduction

Credited with saving millions of lives each year, immunization has proven to be a resounding global health triumph [1]. Yet, despite such tremendous achievements, equitable access remains a critical challenge and progress has stalled in many settings. Tens of millions of infants are under- or un-vaccinated, and communities in marginalized, remote, or fragile settings often lack access to reliable immunization services [2], [3]. Even in countries with relatively stable immunization programs, resource constraints, shifting political priorities, public health emergencies, and vaccine hesitancy threaten continuity and coverage. The COVID-19 pandemic has well illustrated the fragility of routine immunization programs around the globe [4], [5]. However, the initial uptake, demand and roll-out of COVID-19 vaccines in early 2021 shows an exceptional commitment to vaccination as an essential tool to help end the pandemic.

As the global community grapples with the COVID-19 pandemic, the need to prioritize equity and sustainability of immunization has never been more urgent nor apparent. Today, vaccines are available for more than 20 diseases and more are under development; about 86% of infants were vaccinated with the third dose of diphtheria-tetanus-pertussis vaccine (DTP3) in 2019 [1], [6]. Beyond health, vaccination delivers additional social, educational, and economic benefits [7], [8], [9]. The unprecedented pace of COVID-19 vaccine development, production, and now the start of distribution is a testament to the continued immunization gains and progress in combatting vaccine-preventable morbidity, mortality, and the accompanying socioeconomic impacts. Still, the increasing mortality rate with age with the COVID-19 pandemic underlines the Immunization Agenda 2030 (IA2030) tenet of immunization across the life course [10]. Controlling this pandemic requires widespread availability of immunization services and high vaccine acceptance across all ages, yet adults in many countries are hesitant or lack access to this crucial intervention [11].

**Fig. 1:**
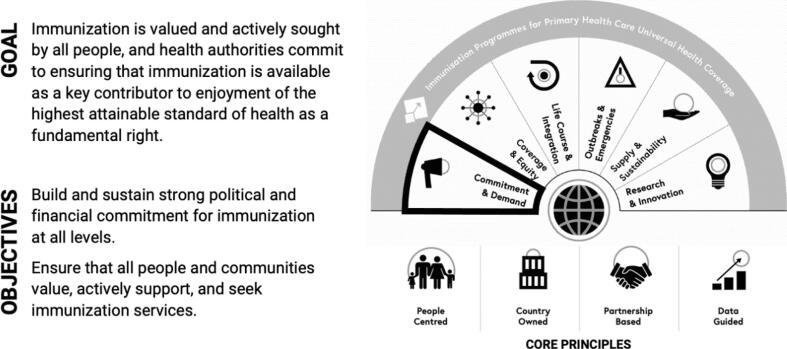
Goal and Objectives for IA2030 Strategic Priority 2 (Commitment & Demand) [6].

To achieve and maintain high immunization coverage and address remaining equity and sustainability challenges, IA2030 identifies commitment and demand as a critical strategic priority (Fig. 1) [6], [12]. Broad commitment is essential to developing, supporting, and sustaining comprehensive and effective immunization and health systems that can deliver vaccines to everyone who needs them, regardless of their location, gender, age, culture, or socioeconomic status. Government commitment and political will are fundamental to IA2030 and required now more than ever with the COVID-19 pandemic and its vaccination campaigns. In addition to government commitment, advocacy and accountability by broad stakeholder coalitions and civil society actors are vital to sustain the resource allocation and technical support needed to coordinate, implement, and evaluate immunization programs and to facilitate policy change, where needed [6], [7], [12].

Policies and programs alone are insufficient to realize sustainable gains from immunization; strong community demand for and uptake of immunization services across the life course are also essential [13]. Immunization programs must consider a range of factors, including: availability and convenience of quality immunization services, caregiver and individual experiences with health service providers, community attitudes and social influences, and people’s understanding of the value and benefits of vaccination [14], [15]. Health services and communication must be tailored to the unique and varied social, cultural, demographic and economic contexts [14], [15]. To build strong acceptance and demand for vaccination among caregivers and their communities, national governments must be committed to delivery of quality services, building trust and engaging communities to ensure that programs are responsive to the needs and perspectives of all people across the life-course.

**Fig. 2:**
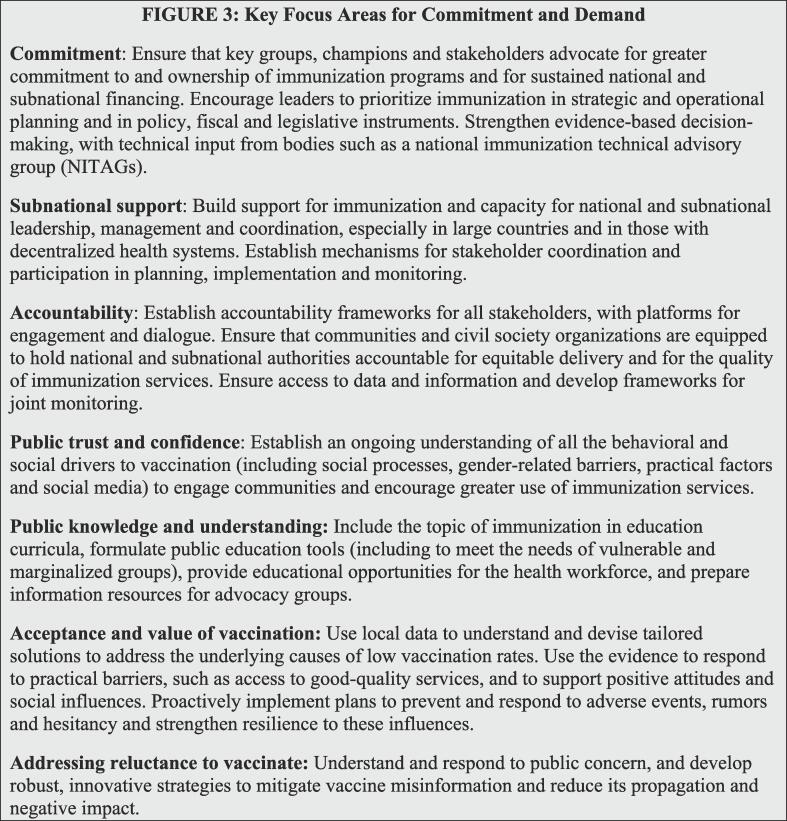
Key focus areas for Strategic Priority 2 – Commitment and Demand [6].

From community members to global leaders, commitment to the overarching vision and goals of IA2030 and demand for quality services will be central to ensuring that immunization is accessible, valued, and actively sought by all people. Building on the agenda’s collaborative development process, interventions to improve commitment and demand aim to be people-centered, partnership-based, country-owned, and data-guided. In this paper, we summarize Strategic Priority 2 [6], which describes key challenges to commitment and active uptake of vaccination and provides recommendations across critical focus areas to strengthen buy-in at all levels (Fig. 2).

The implementation of COVID-19 vaccination programs around the world—including efforts to ensure equitable COVID-19 vaccine supply and access, to recover lost gains in immunization coverage as a result of the pandemic, and to rebuild trust and use of vaccines and immunization systems—will demonstrate the degree to which countries who signed onto the IA2030 Agenda in 2020 are truly committed to ensuring access for all, regardless of age, gender, socioeconomic status, or geographic location.

## 2 Challenges to sustaining commitment and demand for vaccination

Despite being a core component of public health systems worldwide, a range of challenges threaten continued political, financial, and public support for immunization services, not least among them the ongoing COVID-19 pandemic. To effectively shore up commitment to immunization programs and strengthen community and individual demand for immunization services, we note three key challenges: shifting political priorities and resource constraints, visibility of vaccine-preventable diseases, and behavioral and social drivers. Each of these areas will necessitate improved availability and use of quality data to help define the issues and develop targeted, tailored interventions.

### 2.1 Shifting priorities and resource constraints

Across income groups, national and subnational governments are tasked with implementing a range of essential programs, often in the face of constrained resources and shifting donor and financing support. Decision makers must balance complex and often competing political priorities with commitments to existing health programs and must demonstrate that they are meeting the needs of their populations while exercising fiscal judgement. Without continued advocacy and sufficient buy-in from national and subnational stakeholders, immunization coverage targets or commitments may not be met. This is particularly the case when domestic health funds are limited and cannot be (or are not) increased and if funding and resource streams are diverted from immunization to other priorities, as was seen recently when many involved in child immunization were pulled away to support the COVID-19 pandemic response [5], [16], [17].

Evolving global and national political context also has budgetary and financing implications, and thus impacts commitment to immunization along the life-course. Global and national aspirations of universal health coverage and access to immunization are not always met with the necessary political, financial, and technical investments. As co-financing and donor support shift to other priorities or countries transition out of Gavi eligibility as their gross national income rises, thus taking on an increasing share of their vaccine costs, governments must take on a greater share of program and procurement costs [18], [19], [20], [21], [22]. Even changes to funding and capacity support outside of routine immunization have implications. For example, the eventual sunsetting of the Global Polio Eradication Initiative (GPEI) is likely to have cascading financial, human resource, and programmatic implications in countries where GPEI funding has provided substantial support for staffing, program infrastructure, and other resources leveraged across immunization and communicable disease control domains [23], [24]. National and subnational commitment to filling these funding and capacity gaps, and specifically to ensuring access for marginalized or remote populations and maintaining high coverage overall, is crucial to maintaining immunization program gains [25]. Pandemic-driven setbacks in immunization coverage have forced countries and the global community to rethink our approach, looking instead to integration of health services. Sustained commitment is necessary to gather lessons learned from this experience and strengthen programs and policies moving forward, and to strategize how COVID-19-specific resources can and should be reallocated to regain lost ground in routine immunization.

### 2.2 Visibility of disease burden

Infectious disease outbreaks—such as measles, diphtheria, and the ongoing COVID-19 pandemic—present critical challenges to health systems and strain resources; and programs may see human resources and financial support shift from routine programs to emergency response. However, these preventable outbreaks may afford opportunities to strengthen commitment and demand for immunization. Demonstrating the value of vaccination can be more straightforward when disease burden is clearly visible. The urgency and prominence of these emergencies may help propel political action to strengthen primary care services and health systems for both the immediate and longer term, and promote public support for key interventions, like immunization.

Many diseases prevented by vaccination are frequently less visible, often thanks to the success of vaccines and vaccination programs; other vaccines are provided to prevent disease many years in the future. With newer vaccines like those for HPV, various population groups need to be informed about the diseases themselves and prevention efforts. In some cases, names for specific vaccine-preventable diseases do not exist in local languages. These scenarios require greater effort to communicate the risk of disease and the value and benefits of vaccination, cultivate stakeholder support, and ensure that immunization services are easily accessible and appealing to a broader age range. While national governments typically oversee immunization program decision-making and strategic planning, all levels of planning and implementation must work in concert. In decentralized systems, strong commitment, leadership and management capacity, and coordination are often even more critical to ensure efficient, effective, equitable program implementation [26], [27].

### 2.3 Behavioral and social drivers

Immunization programs have traditionally focused on delivery—ensuring the right vaccine, in the right condition and right quantities, reaches the right place at the right time. However, this approach has neglected to give the same weight to the social, cultural and behavioral factors that drive people’s acceptance of and demand for vaccination. Missed opportunities to understand and address these human factors, generate and leverage public support, and build political commitment have the potential to contribute to lack of information, misinformation, and growing vaccine hesitancy in countries around the world [28], [29]. Pockets of vaccine refusal place decades of gains in reducing vaccine-preventable disease morbidity and mortality at risk [30], [31], [32], [33].

Critically, gender-related barriers limit access to, demand for, and impact of immunization services [34]. Immunization programs and policies must account for gender-related factors, which requires going beyond traditional analyses of immunization coverage and recognizing interactions of gender norms with socio-cultural and economic factors that, together, impact the ability of caregivers and communities to access care and health workers to effectively deliver services [35], [36].

The need remains for evidence-informed plans to stimulate confidence in and demand for vaccination, promote the importance of timely and complete vaccination, and leverage local coalitions, advocates, and decision-makers to generate the necessary political commitment for sustainably funded and well-managed programs. Routine immunization programs have largely focused on infants and young children; however, as new vaccines become available across the life-course, including COVID-19 vaccines, and as countries experience demographic shifts, there is a need to update platforms currently in place to deliver vaccines and generate demand throughout the life-course.

At their core, immunization programs depend on reliable, high-quality, local data. Yet, in some settings, lack of local data on the drivers and facilitators of vaccine uptake—or inadequate gender-responsive community engagement to understand priorities—may impede efforts to design, implement, and evaluate targeted interventions to improve immunization acceptance and demand. To close remaining gaps, governments and implementing partners at all levels must collaborate to identify under-vaccinated populations, understand their perspectives, and respond to their needs, both in routine settings and in times of crisis such as the COVID-19 pandemic, by assessing and addressing behavioral and social drivers more systematically [37].

## 3 Recommended interventions

**Fig. 3:**
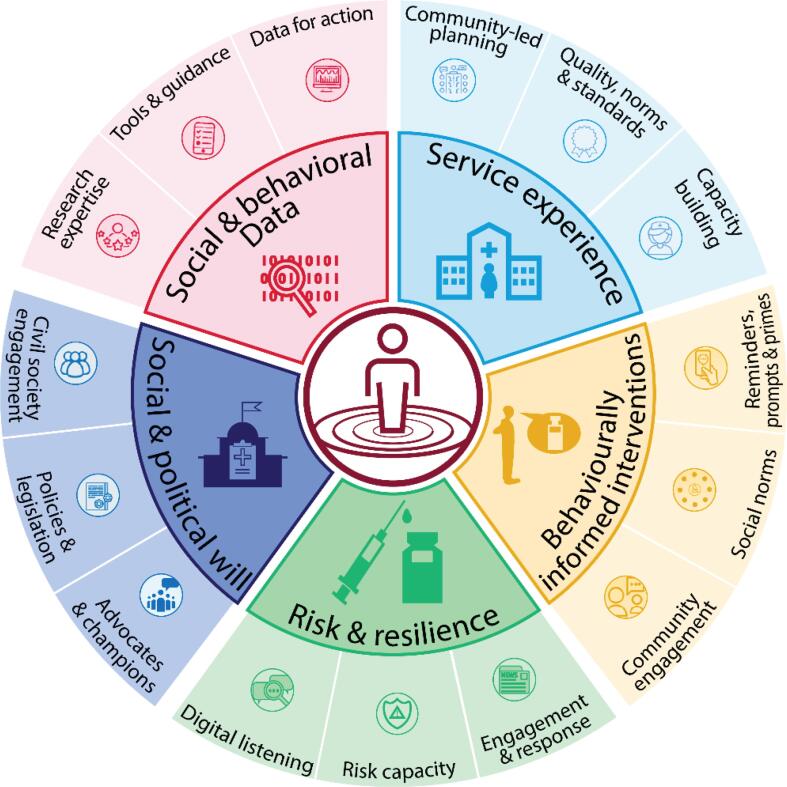
Global demand planning framework, illustrating the five areas that are essential to people-centered programming for resilient demand. Note that each of the five areas are also interconnected, with data cross-cutting all.

Addressing these challenges and sustaining gains against the health, social, and economic impacts of vaccine-preventable diseases require a multi-pronged strategy (Fig. 3). We propose a set of interventions across five interconnected domains: building and sustaining national commitment, supporting subnational leadership and community engagement for immunization across the life course, ensuring accountability at all levels, promoting acceptance and demand for vaccination, and addressing reluctance to vaccinate.

### 3.1 Build and sustain national commitment

**Fig. 4:**
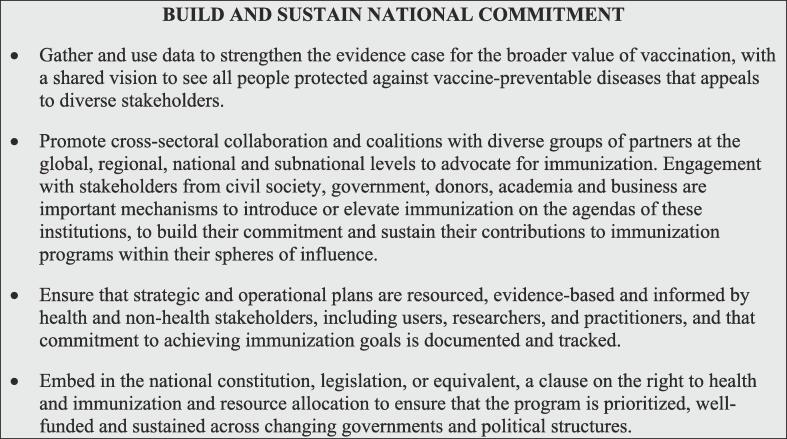
Interventions to build and sustain national commitment.

Government commitment is central to supportive policy, financing, regulatory, and legislative frameworks that apply across the life course [24]. It can be demonstrated by dedicating institutional capacities and financing to immunization, institutionalizing immunization in legislation, creating operational processes that enhance program delivery and improve equity, integrating vaccination into broader essential services across the life-course, or making public pronouncements in support of immunization (Fig. 4). Legal frameworks, such as legislation on the right to immunization, can also help uphold political commitment and ensure accountability, although further research is needed in this area [38], [39].

Data and recommendations from multiple stakeholders inform decision-making and prioritization, and it is important to understand the pathways through which information reaches decision-makers [40]. National Immunization Technical Advisory Groups (NITAGs) play a central role in supporting evidence-based decision-making by national health authorities and tailoring recommendations to fit the unique country context; they also serve as a marker of national commitment to immunization. Because they are independent of the government and centered on leveraging their scienfitic and technical expertise for evidence-driven recommendations, NITAGs can also help to build trust in national decision-making and implementation [41], [42], [43], [44].

Commitment hinges on engagement of government policy and program officials as well as civil society and community leaders, who play essential roles in advocating for political prioritization and action, holding leaders and programs accountable, and amplifying community needs and priorities. The commitment of a wide range of actors to vaccination programs can help support accountable and sustained resource allocation, evidence-informed policymaking, and mobilization of technical assistance to effectively and equitably implement immunization programs. This should include local health departments and service providers, as well as a broad representation of civil society, including professional organizations, academia, community, women’s and youth groups, faith-based organizations, parliamentarians, media, and others, including the private sector.

### 3.2 Support subnational leadership and stakeholder engagement

**Fig. 5:**
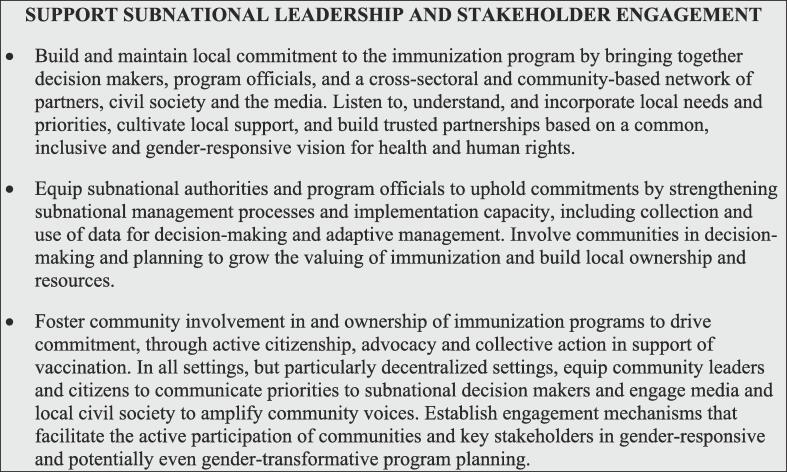
Interventions to support subnational leadership and stakeholder engagement.

While national governments often have the primary responsibility for implementing these programs, commitment must be all-inclusive—built and sustained at the community, district, national, regional and global levels—to close immunity gaps, manage risks, and build the resiliency and sustainability of both immunization programs and community and individual support for them (Fig. 5). In settings where administrative authority is decentralized to state, regional or provincial levels, it is crucial to build commitment not only at the national level but also with subnational authorities and informal leadership [26]. Efforts to strengthen commitment must include a range of stakeholders spanning the health system: ministers of health, finance, education, planning, community development, and other key areas; parliamentarians, as advocates or enactors of legislation; mid-level managers, for program implementation and engagement of the local community; and the health workforce, for their role in healthcare service delivery and the care and attention they contribute daily.

It is essential that quality services are responsive to local community needs and prioritize equity—working to improve health outcomes for all, including the most marginalized and vulnerable. This is particularly true in decentralized systems, where decision-makers are closer to their constituents but may have limited access to national-level resources [27]. Strategies to build subnational commitment must be tailored to the local context, ensure adequate capacity among subnational authorities to implement decisions in response to local needs, and be paired with advocacy and demand generation to promote constituent participation [27].

### 3.3 Establish accountability at all levels

**Fig. 6:**
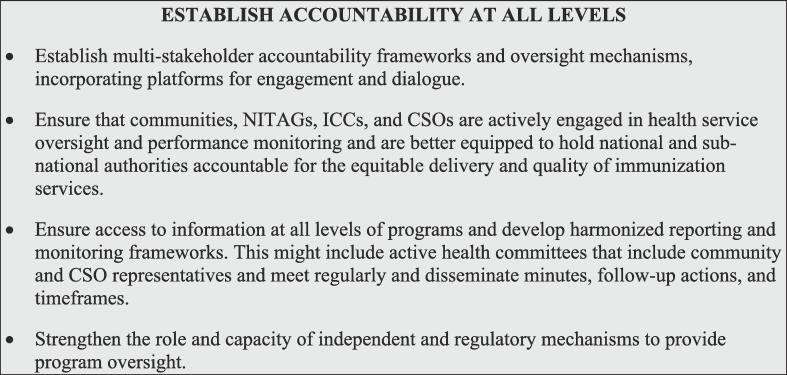
Interventions to establish accountability.

Governance structures and health agencies must accept responsibility and be held accountable for delivering inclusive, quality, and accessible services and for achieving national goals and targets. Achieving accountability requires reliable and transparent information at all levels to help guide action and monitor progress, and to course-correct when necessary (Fig. 6). A range of stakeholders—from global leaders to communities, civil society, scientific experts (e.g., NITAGs), coordinating groups such as the Immunization Interagency Coordinating Committee (ICC), and media—must be empowered to hold national and local authorities and programs accountable for immunization commitments.

Responsibility and accountability exist at many levels, but countries have special obligations to adequately resource and implement plans that systematically address the needs of all vaccine-eligible populations and providers, including people living in marginalized communities. Beyond monitoring vaccination coverage and timeliness, this requires countries to strengthen information systems and incorporate guidance on demand-related indicators (along with other immunization data) to address data gaps and improve registries and processes to serve as a foundation for immunization monitoring [45], [46]. Quantitative and qualitative data help to drive action. Countries and key partners must commit to and conduct routine monitoring of progress towards local and national vaccination targets. Wherever possible, there must be alignment across the health sector with other accountability efforts [47], [48]. The IA2030 commitment and demand efforts are being aligned with the IA2030 Scorecard and incorporated as part of the data working group support that spans across the IA2030 strategic priorities [49].

### 3.4 Promote acceptance and demand

**Fig. 7:**
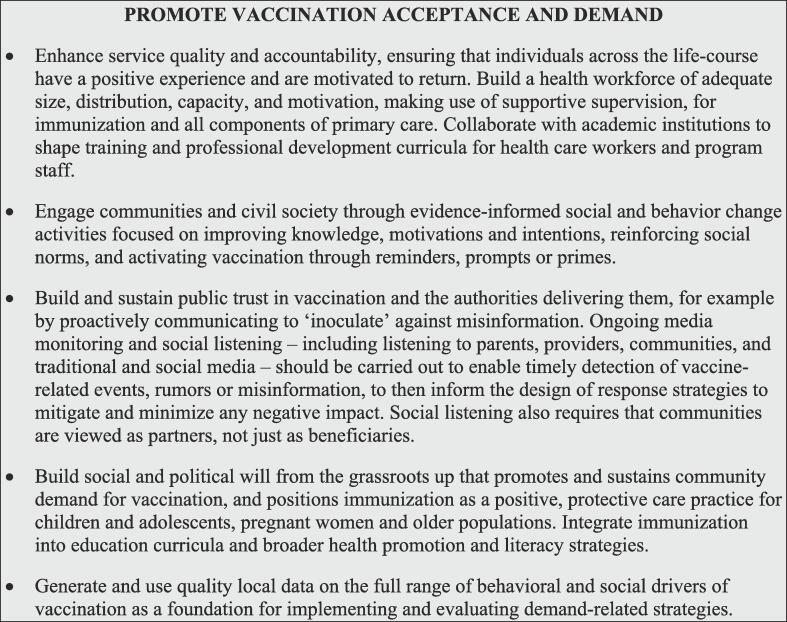
Interventions to promote vaccination acceptance and demand.

Availability of immunization services and commitment to adequate financing and resourcing must go hand in hand with programs to increase access to and demand for vaccination (Fig. 7). Context-specific logistical, economic, and sociocultural challenges, including gender-related barriers, can contribute to uneven, sub-optimal coverage [50]. Health systems sometimes struggle to effectively engage caregivers and communities, leading to weakened acceptance and demand for vaccination, declining coverage rates, and coverage inequities. Demand must be continually fostered by governments, health practitioners, providers, community leaders, and civil society.

Delivering high-quality, convenient vaccination services is key to ensuring positive experiences in vaccination settings across the life-course and can help to improve acceptance and demand by facilitating actions and removing barriers to use of services [51]. Engaging communities in planning and implementing quality services can help reinforce social norms to vaccinate and present vaccination as a social contract [52]. Co-development of a program to better respond to the needs and context of the community is a key ingredient for success.

Building public trust in immunization is essential to ensure community demand, and it hinges on listening to individuals and communities, understanding their fears and concerns and addressing their needs. To increase vaccination acceptance and demand, it is essential to recognize the influence of individual and contextual determinants on vaccination behaviors, including how they access information and their motivations. Now more than ever, with multi-media information sources readily available, authorities must communicate proactively and factually, consider a wide range of channels and trusted spokespeople and social marketing, and have robust coordination mechanisms and response plans in place [53], [54].

### 3.5 Address reluctance and barriers to vaccination

**Fig. 8:**
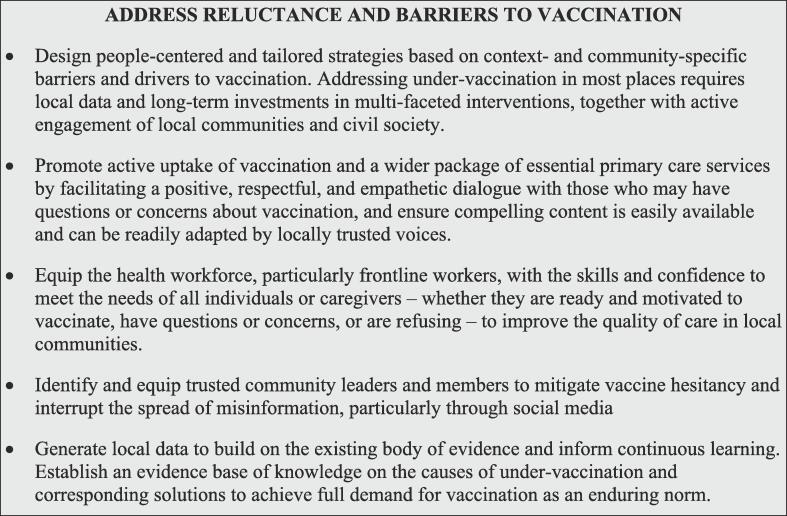
Interventions to address reluctance and barriers to vaccination.

Despite strategies to address barriers to immunization, hesitancy and refusal may remain for a variety of reasons. Strengthening trust in immunization systems and the health system broadly, and improving both individual and program resilience to shocks, can help mitigate the risk of vaccine confidence crises [55], [56]. The reasons for reluctance vary widely within and among countries and communities, driven by a range of supply- and demand-side factors and even differing by vaccine. Understanding these determinants can help inform strategies to address vaccine reluctance and build trust in and uptake of services (Fig. 8).

Health workers are trusted figures and influential in individual and caregiver vaccine decision-making. Countries should train and equip frontline workers to have productive and interactive immunization-related conversations with parents, caregivers, and individuals, listening and acknowledging concerns in a respectful and empathetic manner and providing accurate information about the value of vaccination; critical to this effort is providing appropriate information to health workers to address their own questions or hesitations about vaccination. There is a body of evidence that supports making vaccination an easy, convenient and default option, enhanced by strong relations and trust between patients/caregivers and health workers [51], [54].

Other trusted community voices can also be influential in community norms and vaccination uptake. Civil society organizations, faith-based leaders, and community leaders can play a key role in building trust in immunization by channeling information on public needs, priorities, and concerns to relevant authorities and programs, and facilitating interactions between communities and service providers.

People-centered methodologies, such as human-centered design, can help to inform understanding of the gender, geographic, or economic barriers faced by individuals and caregivers to accessing services. Lack of affordable transport to and from health facilities, inconvenient clinic hours or location, high cost of vaccines or health care, unavailable facility or outreach sessions, and service disruptions can all influence reluctance to vaccinate. Fortunately, relatively simple, low-cost strategies like transport vouchers, changes in facility hours to suit parents and individuals, and mobile phone reminders for return dates can all help to address some of these barriers and improve acceptance; improvements to vaccine supply management and forecasting can help to ensure individuals and caregivers seeking vaccination services are met with available supply.

Misinformation, often spread through social media, has become a critical issue for health authorities and communities [57], [58], [59], [60], [61]. Building trust in immunization programs, understanding how specific groups use social media, and providing accurate and audience-tailored information through channels and influencers whom community members trust are crucial to interrupt misinformation spread. Strategies to monitor rumors, misinformation, and disinformation both online and in the community can help inform tailored messages to limit their spread and mitigate hesitancy.

Countries and implementing partners at all levels must assess and address behavioral and social barriers, as well as structural barriers, and recognize and capitalize upon key community assets to leverage trusted voices and build resilience. Programs must dig deeper to truly understand why specific populations have not had their needs met or may be reluctant to accept vaccination, potentially navigating challenging political, social or cultural issues.

## 4 Conclusion

Strengthening commitment to sustainable, equitable programs and promoting demand for vaccination services are crucial to maintain and increase the progress made through immunization to date. Health officials and partners must recognize the drivers of program success and public trust at all levels.

With strong leadership and commitment, health systems can deliver vaccination in a quality, equitable and sustainable manner, but only if critical resources—including human resources and funding for vaccines and annual and long-term program operating costs—are assured. In countries where there are inadequate or disrupted immunization services or primary health care systems, the first priority must be to build the necessary infrastructure and strengthen resilience to overcome shocks.

People must be at the center of immunization programs. Meaningful community engagement will enable more effective program planning and implementation, and help to establish vaccination as a social norm [54], [62]. Social listening and rumor tracking must be leveraged to contribute to understanding drivers of immunization acceptance and to rapidly identify and combat fast spreading misinformation.

Failing to recognize the far-reaching benefits of immunization and substantial return on investment can threaten program sustainability, as policy makers grapple with competing priorities and fiscal challenges. Civil society, community leaders, social networks, caregivers, and individuals all play important roles in vaccination uptake and confidence-building and serve as powerful advocates to exert pressure on decision-makers to improve vaccine availability for all across the life course, including the most vulnerable. As we implement IA2030, countries and partners must be equipped to gather reliable, high-quality data on immunization program status and drivers of community demand, and to employ human-centered design and people-centered approaches that enhance and build on traditional approaches and improve commitment at all levels.

## CRediT authorship contribution statement

**Folake Olayinka:** Conceptualization, Writing – original draft, Writing – review & editing, Project administration, Supervision. **Molly Sauer:** Writing – original draft, Writing – review & editing, Project administration. **Lisa Menning:** Conceptualization, Writing – original draft, Writing – review & editing. **Diane Summers:** Conceptualization, Writing – original draft, Writing – review & editing. **Chizoba Wonodi:** Conceptualization, Writing – original draft, Writing – review & editing. **Susan Mackay:** Conceptualization, Writing – original draft, Writing – review & editing. **Noni E. MacDonald:** Conceptualization, Writing – original draft, Writing – review & editing. **J. Peter Figueroa:** Conceptualization, Writing – original draft, Writing – review & editing. **Benjamin Andriamitantsoa:** Conceptualization, Writing – original draft, Writing – review & editing. **George Bonsu:** Conceptualization, Writing – original draft, Writing – review & editing. **Pradeep Haldar:** Conceptualization, Writing – original draft, Writing – review & editing. **Ann Lindstrand:** Conceptualization, Writing – review & editing. **Lora Shimp:** Conceptualization, Writing – original draft, Writing – review & editing, Supervision.

## Declaration of Competing Interest

The authors declare that they have no known competing financial interests or personal relationships that could have appeared to influence the work reported in this paper.

## Data Availability

No data was used for the research described in the article.
